# Differences in the Awareness and Knowledge of Radiological and Nuclear Events Among Medical Workers in Japan

**DOI:** 10.3389/fpubh.2022.808148

**Published:** 2022-03-30

**Authors:** Kanae Ochiai, Tomo Oka, Nagisa Kato, Yuji Kondo, Yasuhiro Otomo, Raymond E. Swienton

**Affiliations:** ^1^Trauma and Acute Critical Care Medical Center, Tokyo Medical and Dental University Hospital of Medicine, Tokyo, Japan; ^2^Emergency Medical Center, Fujisawa City Hospital, Kanagawa, Japan; ^3^Department of Emergency, Japanese Red Cross Medical Center, Tokyo, Japan; ^4^University of Texas Southwestern Medical Center, Dallas, TX, United States

**Keywords:** radiological, nuclear, training, education, preparedness, occupation

## Abstract

**Background:**

Previous research revealed a lack of comfort and knowledge regarding nuclear and radiological events among medical staff. We investigated the awareness and knowledge of radiological and nuclear events among the Japanese medical staff by comparing differences by occupation (doctors, nurses, and other medical specialists).

**Methods:**

We conducted a cross-sectional questionnaire survey among trainees undergoing Japanese disaster medical training courses between July 2014 and February 2016. The differences by occupation were evaluated for all questions on awareness and knowledge concerning disasters or radiological and nuclear events and demographics.

**Results:**

Among the occupations, there were significant differences in the willingness to work onsite based on the types of disaster, familiarity with the national disaster medical response system, the accuracy rate of some knowledge about medical practice and the risk, and demographic characteristics such as practical experience and educational degree. The accuracy rates of responses to some questions on knowledge were very low in all occupations.

**Conclusion:**

There were significant differences in awareness and knowledge of radiological and nuclear events by occupation. We believe that the results can be used to develop and modify the content of training courses on radiological and nuclear events to make such courses beneficial for each healthcare worker.

## Introduction

Although radiological and nuclear events are rare, preparedness for such events is necessary because they can cause serious damage. Physical and mental health issues have been long-term problems in the aftermath of nuclear accidents in Fukushima and Chernobyl (1–3). Moreover, the Fukushima nuclear disaster caused severe damage to the economy, as industrial shipments from Fukushima fell to 85% in 2011 (1). It also gave rise to social problems such as discordance in families and communities (1). Therefore, in recent years, many countries have put in place preparedness for chemical, biological, radiological, nuclear, and explosive (CBRNE) disasters. Such preparedness includes the construction of a network of biological dosimetry (4), investigation of the equipment or systems required for CBRNE disasters at hospitals and emergency medical services (5–7), and assessment of education and training for CBRNE disaster response (8–10). The recent coronavirus disease 2019 (COVID-19) pandemic also has the characteristics of a mass casualty, and intensive care unit (ICU) preparedness to adequately address it became an urgent global concern. Therefore, several guidelines for planning and roadmaps that enable ICU preparedness have been established (11–15). The COVID-19 pandemic was too intense to manage, and most healthcare providers realized the importance of preparedness. Efficient educational methods relevant to CBRNE disasters are needed.

Japan has experienced some major radiological and nuclear events, such as the Fukushima No. 1 nuclear power plant accident and the nuclear bomb detonations in Hiroshima and Nagasaki (16). Japanese people are aware of the dangers of radiological and nuclear events because there are 60 nuclear plants across the nation, and 8 of these were in operation as of December 2021 (17), despite this country being seismically active. Japanese also have anxiety about radiological and nuclear events based on the Fukushima nuclear power plant accident experience and their knowledge of the history of nuclear bombings.

Dallas et al. (18) conducted a questionnaire survey to compare American and Japanese medical personnel's willingness to respond to, their familiarity with, and their relevant knowledge of, radiological and contamination risks; they found that most responders were still very uncomfortable with radiological and nuclear events. The survey targeted people who participated in various medical and disaster conferences and courses in the USA and Japan. Compared to US respondents, Japanese respondents were less likely to be willing to work in a dirty bomb scenario (−27%) or treat casualties at their hospital (−5.3%), respectively. Concurrently, they indicated that Japanese respondents had insufficient knowledge about these events, such as knowledge about personal protective equipment (PPE) or internal radioactive contamination.

In Japan, the disaster medical assistance team (DMAT), which consists of doctors, nurses, and logistics personnel, is a nationwide medical system that is activated when large-scale disasters occur. Although there are other disaster response systems that consist of registered members, DMAT contains the largest number of registrants. The DMAT training course was modified following the Great East Japan Earthquake (19); however, it does not contain educational content for CBRNE disasters. This is because activities that need to be performed during CBRNE disasters are distinctive and accompanied by significant hazards for general healthcare workers. Therefore, it would be effective to separate the training program for CBRNE disasters from the normal disaster training course and to target medical staff who are knowledgeable about the medical team's activities during normal large-scale disasters (20). Based on the different occupations, the DMAT training course includes separate programs for doctors, nurses, and logistics personnel because of differences by occupation in roles at disaster sites and the educational basis (19). There might also be some differences in the level of awareness or knowledge of CBRNE disasters by different occupations.

Therefore, we conducted a survey of the Japan DMAT training course trainees using the same anonymous questionnaire that Dallas et al. (18).

This survey aimed to determine the level of awareness and knowledge of radiological and nuclear events of medical staff with interest in disaster medicine who were able to be the main member to work in such disasters in Japan. We also aimed to investigate the differences between doctors, nurses, and other specialists. Moreover, we also investigated suggestions for improvement of advanced CBRNE disaster education and training courses, considering the different backgrounds of the medical workers.

## Materials and Methods

We conducted a cross-sectional questionnaire survey. In this survey, paper questionnaires were distributed to trainees who participated in the Japan DMAT training course in Tokyo (between July 2014 and February 2016) and Hyogo (between September 2014 and February 2016). The trainees were hospital medical staff who intended to work at disaster sites during large-scale disasters. Doctors and nurses with any specialty could participate in this course. Logistics personnel included hospital clerks, pharmacists, radiologists, and other medical professional technologists. The questionnaires were distributed during the course and collected until the end of each course.

The questionnaire, which was the same Japanese version used in the previous survey (18), contained 22 questions divided into four sections: 1) willingness to manage exposed casualties, 2) familiarity—local and country disaster system, 3) familiarity—radiological and nuclear contamination risks, and 4) demographic and practice description. Five questions were about willingness to work during CBRNE disasters or familiarity with such disasters, six were to verify respondents' knowledge about radiological and nuclear contamination, and 11 were about respondents' background and demographic characteristics. The English version of this questionnaire is shown in the previous article (18) and the Supplementary Material.

The Japan DMAT secretariat implemented the distribution and collection of the questionnaires. The data obtained from the questionnaires were analyzed at the Department of Acute Critical Care and Disaster Medicine, Tokyo Medical and Dental University.

To evaluate the differences by occupation, the Kruskal-Wallis test was used for continuous variables and the chi-square test for categorical variables. If there were significant differences on univariate analysis, binary logistic regression analysis adjusted for year of birth, sex, and radiation/nuclear experience was conducted for the question items. A *P* < 0.05 was considered statistically significant. All analyses were performed using IBM SPSS Statistics for Windows, version 25 (IBM Corp., Armonk, N.Y., USA).

The survey was approved by the Institutional Review Board (IRB) of the University of Texas Southwestern Medical Center, which reported the previous survey (18). The IRB determined on November 18, 2013, that this survey was exempt in accordance with 45 CFR 46.101(b) (IRB number: STU 082013-073).

## Results

During the survey period, 1,620 questionnaires were distributed during the Japan DMAT training courses. We obtained responses from 904 respondents (55.8%). Since we excluded respondents whose type of occupation (doctor, nurse, or logistics) data were missing, 774 responses (47.8%) were finally analyzed. Regarding occupation, 342 (44.2%), 284 (36.7%), and 148 (19.1%) of respondents were doctors, nurses, and logistics personnel, respectively.

Demographic and background data are shown in Table 1. Significant differences were observed among occupations in terms of year of birth (*p* < 0.05) and practical experience of disasters or public health emergencies; the doctors were the most experienced (*p* < 0.01), and the nurses were the least experienced (*p* < 0.05). There were significantly fewer male nurses (*p* < 0.01), and nurses were the least affiliated with a disaster medical response team (*p* < 0.01). Doctors were most likely doctorate holders (*p* < 0.01). Logistics personnel who participated in the Japan DMAT training courses rarely belonged to a university (*p* < 0.01), but they tended to complete multiple disaster training courses (*p* < 0.05). No significant differences were found in other background data by occupations. The rate of respondents who had experience in the radiation/nuclear science field was 16.8% (doctor 16.7%, nurse 15.8%, and logistics 18.9%).

**Table 1:** Respondents' demographic and background data.

		**Occupation**		
		**Doctor (*n* = 342)**	**Nurse (*n* = 284)**	**Logistics (*n* = 148)**
Year of birth, median (IQR)		1977 (1970, 1982)	1980 (1975, 1984)	1982 (1977, 1986)
Sex, *n* (%)	Male	283 (84.9)	129 (45.7)	135 (91.2)
Country of medical practice, n (%)	Japan	342 (100.0)	284 (100.0)	148 (100.0)
Highest educational degree, n (%)	Undergraduate	6 (1.8)	8 (2.8)	3 (2.0)
	Graduate	159 (46.5)	120 (42.3)	113 (76.4)
	Doctorate	135 (39.5)	10 (3.5)	11 (7.4)
	No response	42 (12.3)	146 (51.4)	21 (14.2)
Type of medical practice, n (%)	Clinic	1 (0.3)	0 (0.0)	0 (0.0)
	Hospital	267 (78.1)	227 (79.9)	126 (85.1)
	University	70 (20.5)	55 (19.4)	15 (10.1)
	Other	3 (0.9)	1 (0.4)	3 (2.0)
	No response	1 (0.3)	1 (0.4)	4 (2.7)
Experience of radiation/nuclear science field, n (%)	Yes	57 (16.7)	45 (15.8)	28 (18.9)
Field of specialty, n (%)		Emergency 130 (38.0)		Clerk 27 (18.2)
		Surgical 126 (36.8)		Radiologist 22 (14.9)
		Internal 43 (12.6)	NA	Other technologists 74 (50.0)
		Radiology 5 (1.5)		Unknown 25 (16.9)
		Other 38 (11.1)		
Affiliation with some type of disaster response team, n (%)	Yes	218 (63.7)	149 (52.5)	95 (64.2)
Experience of responding to a disaster or emergency, n (%)	Yes	76 (22.2)	39 (13.7)	24 (16.2)
Number of training courses completed, n (%)	0	302 (88.3)	258 (90.8)	138 (93.2)
	1	30 (8.8)	17 (6.0)	8 (5.4)
	2–4	7 (2.0)	7 (2.5)	1 (6.8)
	5 or more	0 (0.0)	0 (0.0)	1 (6.8)
	No response	3 (0.9)	2 (0.7)	0 (0.0)

*IQR, interquartile range; NA, not applicable*.

**Figure 1:**
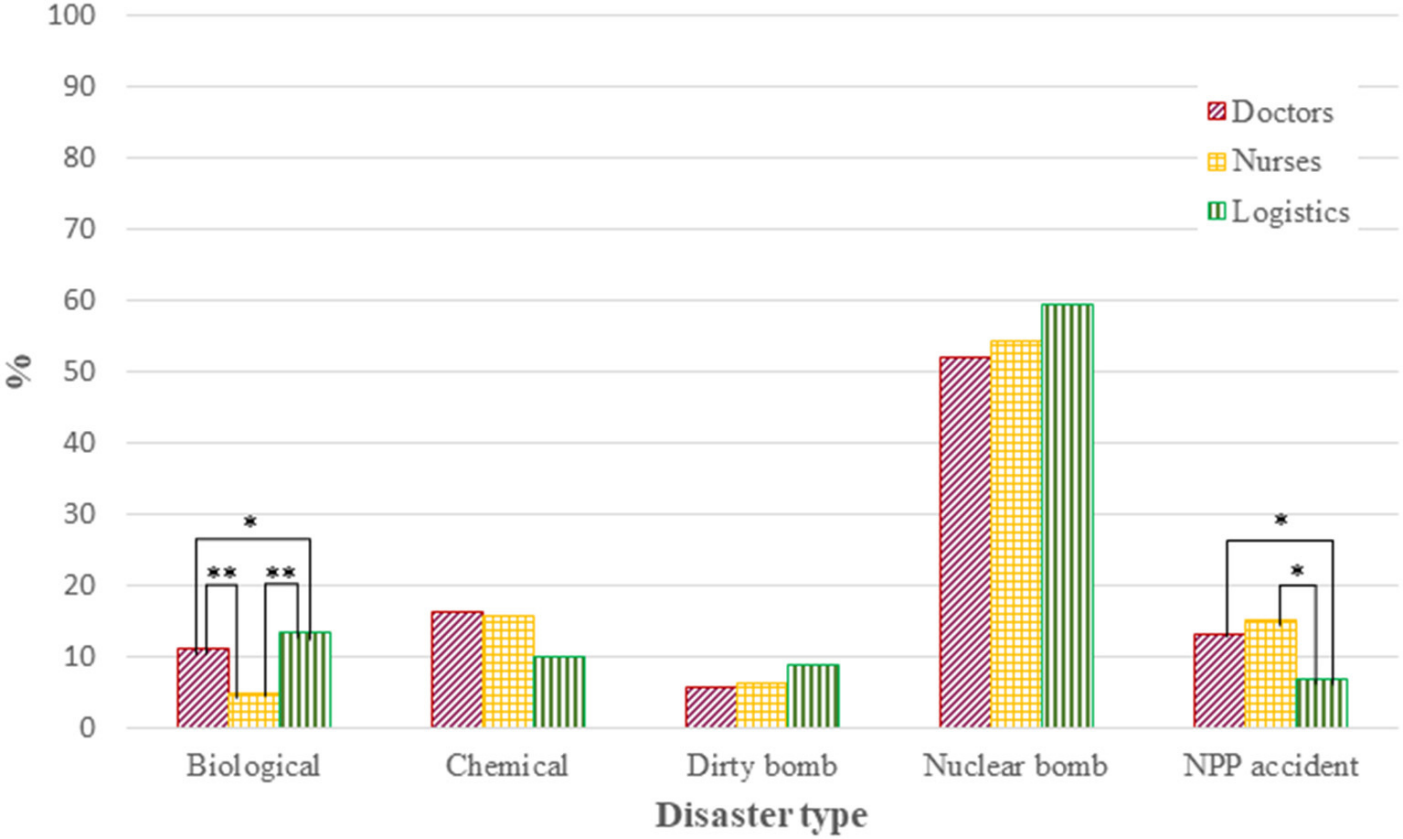
The most important event that may prevent the respondent from coming to work. ^*^p < 0.05, ^**^p < 0.01. NPP indicates nuclear power plant. Chart showing response to Question 1.

**Figure 2:**
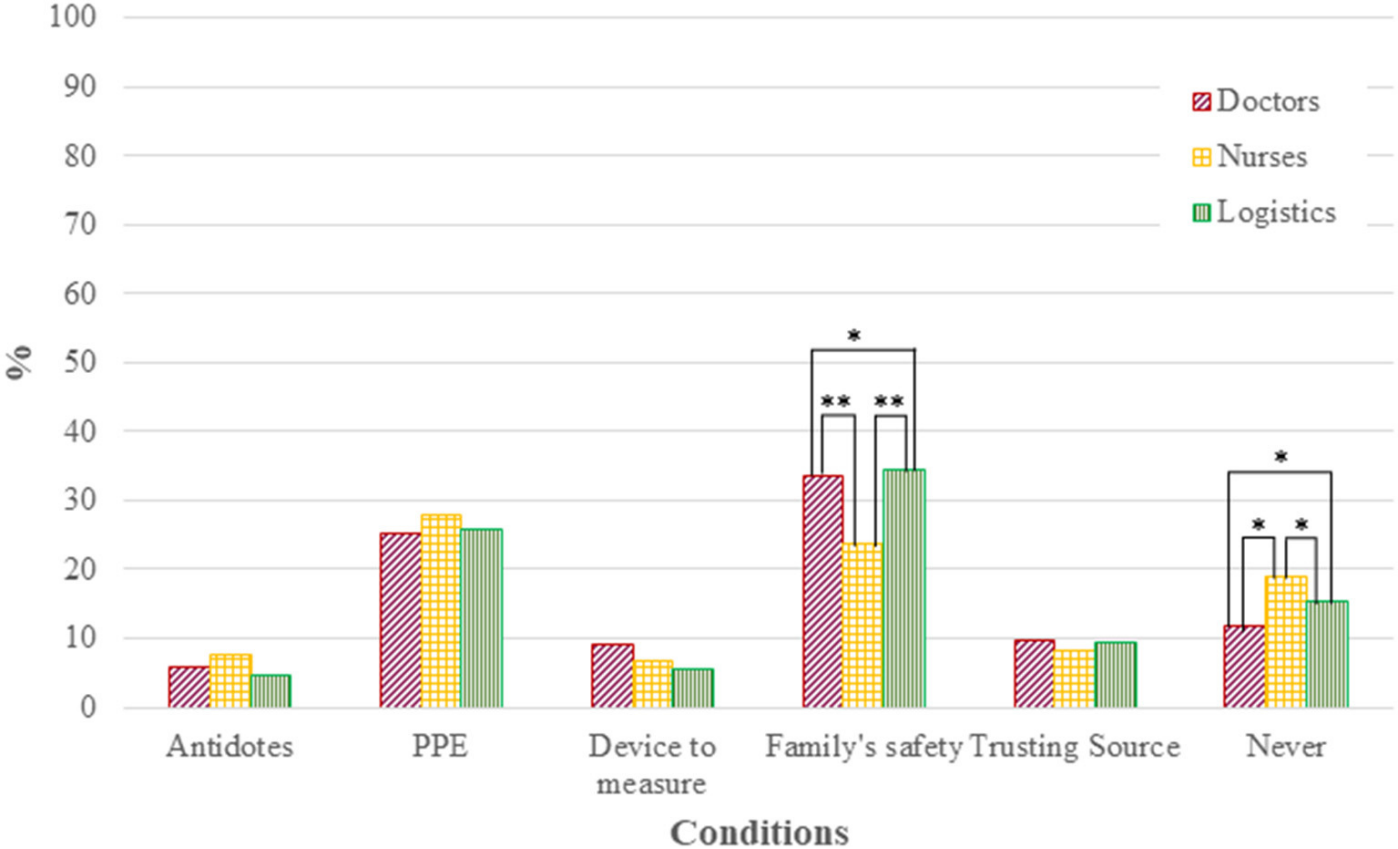
Conditions for willingness to come to work in the event of a nuclear detonation. ^*^p < 0.05, ^**^p < 0.01. PPE ndicates personal protective equipment. Chart showing response to Question 4.

The most important disaster type that may prevent medical staff from coming to work was nuclear bomb detonation for all occupations (Figure 1). The percentage of nurses who consider biological disasters the most important event preventing them from going to work was lower than those of other occupations (*p* < 0.01). Logistics personnel tended to consider that the nuclear power plant accident was not so important to prevent them from coming to work (*p* < 0.05). The most important condition for medical staff to be willing to come to work after a nuclear detonation was their family's safety, except for nurses (*p* < 0.01; Figure 2). Some respondents never intended to work after a nuclear detonation, and the rate of unwillingness was the highest among nurses (*p* < 0.05). However, the significant differences observed in univariate analysis were diminished on binary logistic regression analysis. All occupations preferred to work at the hospital they belonged to rather than at the disaster site under the possibility of radio-nuclear contamination (Supplementary Figure 2).

**Figure 3:**
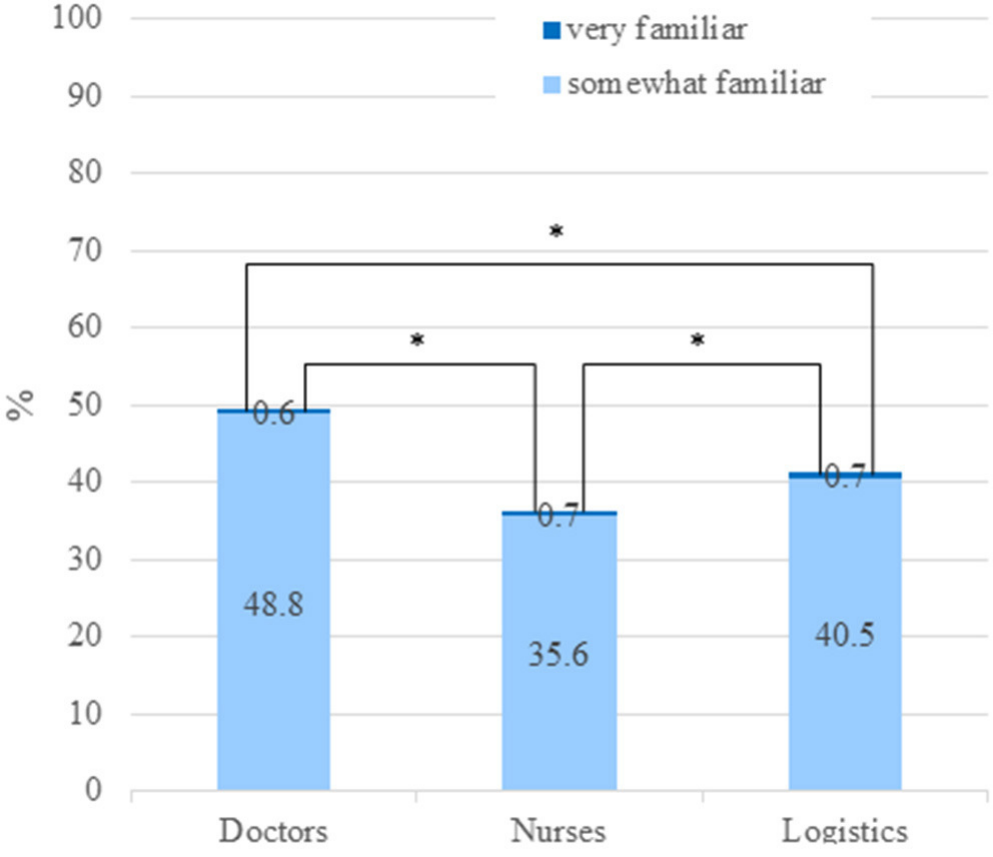
Familiarity with disaster response teams. ^*^p < 0.001. Chart showing response to Question 6.

The results of familiarity with the disaster medical response team are shown in Figure 3. There were significant differences among the occupations regarding familiarity, and nurses were less familiar with the disaster response team than other occupations (*p* < 0.01) per univariate analysis. After binary logistic regression analysis, sex was the only influencing factor on familiarity (*p* = 0.002, OR 1.796 [1.244–2.593]).

**Figure 4:**
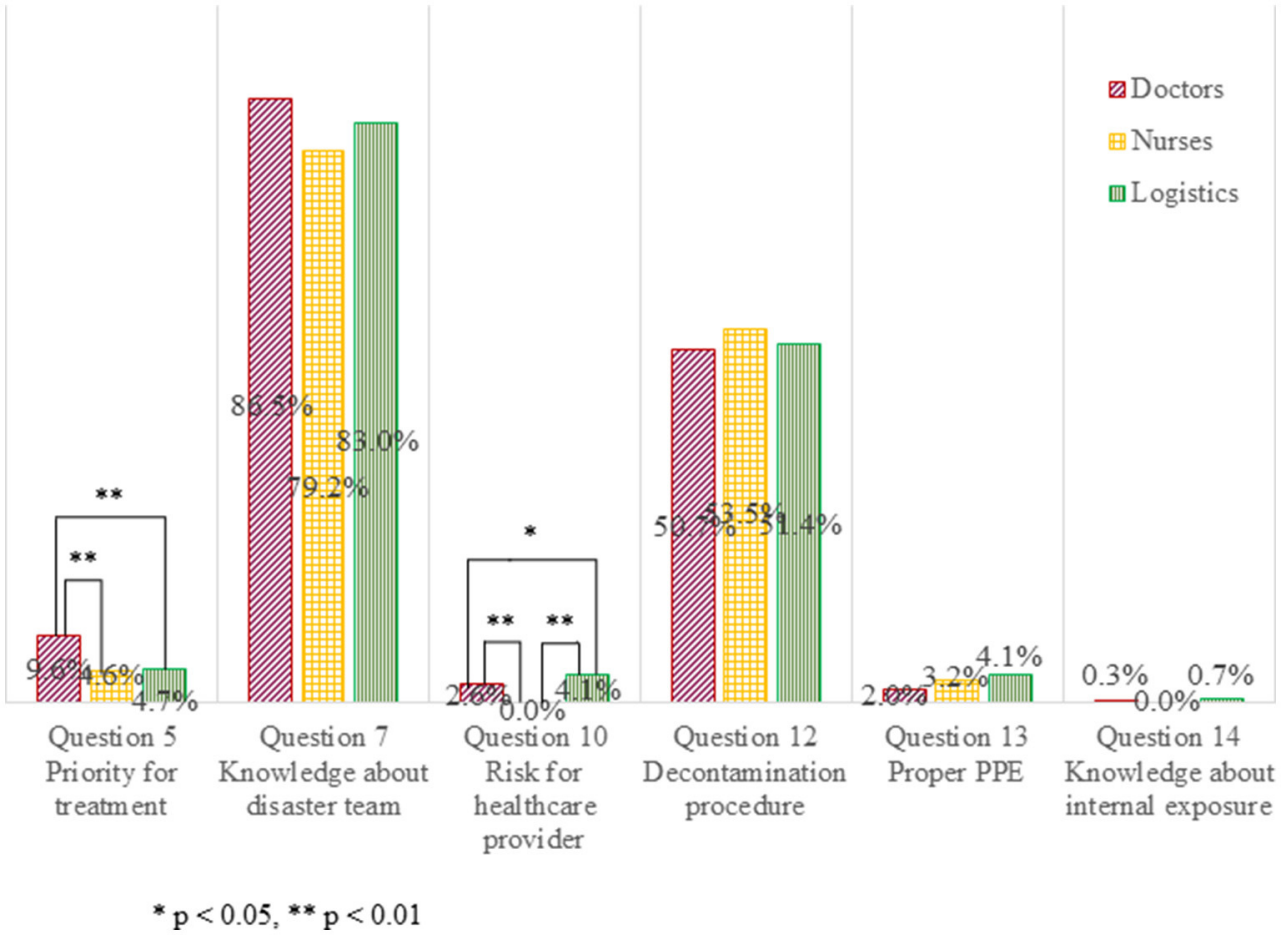
Accuracy rate of responses about the respondent's knowledge of the medical practice and the risk.

The highest accuracy rate about the respondents' knowledge was obtained on the disaster team present in Japan for all occupations, from 79.2, 83.0, and 86.5% among nurses, logistics personnel, and doctors, respectively (Figure 4; Question 7). About half of all respondents provided accurate responses about decontamination procedures needed for patients with trauma emergencies or burn injuries under the situation of a nuclear detonation (Question 12). However, the accuracy rates for the other questions were extremely low. There were significant differences in accuracy rate among occupations when the respondents were required to select the highest priority patient who needed treatment (Question 5; *p* < 0.01), and about the number of healthcare providers who were put at risk by treating patients contaminated with radiological material in all radio-nuclear events since World War II (Question 10; *p* < 0.01) per univariate analysis. Doctors as occupation tended to influence the accuracy rate for Question 5 (*p* = 0.062, OR 2.280 [0.959–2.725]) and nurses as occupation influenced the accuracy rate for Question 10 (*p* = 0.007 OR 0.092 [0.016–0.529]) on binary logistic regression analysis. The accuracy rates of responses on proper PPE (Question 13) and internal exposure (Question 14) were very low, but no significant differences occurred among occupation groups.

## Discussion

We conducted a questionnaire survey of participants in the Japan DMAT training course. Our analysis revealed some differences in respondents' awareness and knowledge by occupation.

All occupations were unwilling to work during nuclear bomb explosions. In situations of radiological and nuclear events, family safety and availability of appropriate PPE were the important conditions for respondents to attend work. Some respondents never intended to engage in radiological and nuclear events even if these conditions were fulfilled. Male respondents were more familiar with the disaster medical response team, and there were no significant differences among occupations after adjusting for year of birth, sex, and radiation/nuclear experience. The occupation tended to influence the accuracy rate of some questions that required knowledge in this study. Background of the occupations might be some of the reasons for their accuracy of knowledge. This study could not demonstrate relevance between the knowledge and willingness and risk perception because few questions required straight answers. In a cross-sectional online questionnaire survey of nuclear medicine technologists, Van Dyke et al. reported that attending radiological preparedness training in the last 5 years was significantly associated with increased willingness to respond to radiological and nuclear events (8). Sheikh et al. conducted a cross-sectional online survey of emergency medicine residents and their faculty; they found that physicians who had received training were significantly more knowledgeable and felt significantly more comfortable in caring for victims, performing decontamination procedures, diagnosing and managing acute radiation syndrome and internal contamination, and using detection equipment in radiological disasters (9). The respondents in this study comprised doctors, nurses, and logistic personnel in various specialties, and a low rate of them had experience in the radiation/nuclear science field. Moreover, these respondents answered the questionnaire during disaster medical training courses, and their awareness and willingness of radiological and nuclear events might be improved. It demands caution to compare the results of this study with those of other previous studies because the backgrounds of subjects and research methods were different. Nevertheless, periodic training and knowledge updates are needed to increase the willingness of medical staff to respond to radiological and nuclear events.

There was a similar tendency of awareness for a biological disaster such as a pandemic to a radiological and nuclear event. In the recent COVID-19 pandemic, several survey results revealed that approximately 61–93% of nurses were willing to participate in the care of patients with COVID-19 (21, 22). The safety of family and oneself, training, communication, and compensation are the major concerns for healthcare workers to attend work under the COVID-19 and other pandemic situations (21–24). These tendencies are consistent with the results of our survey.

The accuracy rate for some questions about respondents' knowledge was low in this survey. A few respondents answered practical questions correctly, such as determining treatment priority (Question 5), selecting proper PPE (Question 13), and treating trauma patients with internal radioactive contamination (Question 14). In addition, respondents rarely chose the correct answer for the question on historical knowledge about the number of healthcare providers who were put at risk by treating contaminated patients in all radio-nuclear events since World War II (Question 10). In particular, incorrect responses tended to be over-protective and over-fearful, such as selecting a higher level of PPE (Question 13), indicating higher perception in treating patients with internal radioactive contamination (Question 14), and estimating a larger number of healthcare providers who were put at risk while engaging in radiological and nuclear events (Question 10). There were differences among occupations in the accuracy rates for Questions 5 and 10, although the accuracy rate was low in all occupations. Dallas et al. suggested that Japanese respondents (61%) were much more likely to state that they did not know what type of PPE was needed for radioactive contamination than American respondents (15%) (18). Likewise, for the question about the perception of their risk in treating patients with internal radioactive contamination, an overwhelming majority of Japanese respondents indicated that they did not know, and they tended to estimate a higher risk of radioactive exposure (18). These misconceptions might be attributed to Japanese people's fearfulness regarding radiological and nuclear events based on the experiences of the Fukushima nuclear power plant accident (1) or knowledge of the history of nuclear bombings in Japan (16). Therefore, a training program for such disasters is needed to provide precise knowledge and eliminate anxiety.

Education on disasters involving radiological and nuclear events or CBRNE disasters is a pressing issue. A previous study by Sheikh et al. (9) indicated that respondents preferred packaged educational materials, classroom teaching at the workplace, drills, and case-based scenarios rather than online training and classroom teaching at a location other than the workplace. They also suggested that knowledge gaps in these areas could be due to reasons such as unappealing training formats, incomplete or limited availability of radiation-response training, or lack of opportunity for hands-on training with radiation detectors. Blumenthal et al. proposed a training strategy with members of the healthcare delivery system classified into four tiers, with tasks identified for each tier, along with the radiation-relevant knowledge needed to perform these tasks (10). This strategy is similar to that of the Japan DMAT training course, which includes separate programs by occupation (doctors, nurses, and logistics personnel) (19). It is controversial, and more investigations are expected to determine which type of training course is better to achieve a high educational effect. However, the separate program for each group divided by background or experience might elevate the motivation and comprehension level.

Both medical and technical knowledge (toxicology, biology, and radiology) are needed to engage CBRNE disaster events as medical team members. Our research suggests that background knowledge may be different between occupations. Therefore, it is reasonable to prepare separate training programs for each occupation, to improve the knowledge level of each.

The present survey had some limitations. First, it was a self-report questionnaire survey. However, the questionnaire contained questions that required the respondents to select clear distinctive alternatives that were specific, unlike questionnaires with responses on a Likert scale where the alternatives are based on subjectivity, making it difficult to interpret the reasons for the choice of responses. Second, the response rates of this survey were 55.8% (904/1,620) and 47.8% (774/1,620) for initial responders and those included in the final analysis, respectively. Moreover, because the paper questionnaire was distributed, it was difficult to check blanks in answer columns for each question, and 14.4% of respondents were lost to a missing indication of occupation. The online survey might be more suitable for pointing out no answers and useful for increasing the response rate. Despite the low response rates, the 774 respondents included in the study constituted a sufficient number for the analysis. Third, since this survey targeted medical staff who underwent a disaster training course and intended to work at disaster sites, the cohort is considered a highly motivated group for disaster medicine and CBRNE events. Therefore, this cohort could be biased. It requires caution to interpret the result of this study and to compare it with previous studies because the questionnaire was distributed during the training course, and the awareness and knowledge were improved more than usual or that at baseline. Nevertheless, our subjects were the main candidates who are engaged in such disaster events, and the results of this survey are useful to modify the curricula of the current stratified type of disaster training course in Japan.

In conclusion, our survey revealed differences in levels of knowledge of radiological and nuclear events by healthcare occupation. Japanese people's fearfulness regarding radiological and nuclear events based on the experiences of the Fukushima nuclear power plant accident or knowledge of the history of nuclear bombings in Japan may explain the results of this research. The differences might be based on the educational background of each occupation. The results can be useful in the development and improvement of training courses for managing radiological and nuclear events. The development of a course that could compensate for the lack of knowledge of each healthcare worker would be beneficial.

## Data Availability Statement

The raw data supporting the conclusions of this article will be made available by the authors, without undue reservation.

## Ethics Statement

The studies involving human participants were reviewed and approved by Institutional Review Board (IRB) of the University of Texas Southwestern Medical Center. Written informed consent for participation was not required for this study in accordance with the national legislation and the institutional requirements.

## Author Contributions

KO designed the study, analyzed the data and was a major contributor in writing the manuscript. TO, NK, YK, YO, and RS designed the study and revised manuscript. All authors participated in the discussion about the interpretation of data. All authors contributed to the article and approved the submitted version.

## Conflict of Interest

The authors declare that the research was conducted in the absence of any commercial or financial relationships that could be construed as a potential conflict of interest.

## Publisher's Note

All claims expressed in this article are solely those of the authors and do not necessarily represent those of their affiliated organizations, or those of the publisher, the editors and the reviewers. Any product that may be evaluated in this article, or claim that may be made by its manufacturer, is not guaranteed or endorsed by the publisher.

## Abbreviations

CBRNE: Chemical, Biological, Radiological, Nuclear and Explosive
COVID-19: Coronavirus disease 2019
DMAT: Disaster medical assistance team
ICU: Intensive care unit
IRB: Institutional Review Board
PPE: Personal protective equipment.
