# Supplementary figures and images for: Differences in the Awareness and Knowledge of Radiological and Nuclear Events Among Medical Workers in Japan
Source: Front Public Health. 2022 Mar 30;10:808148. doi: 10.3389/fpubh.2022.808148 (PMC9006773; doi:10.3389/fpubh.2022.808148)

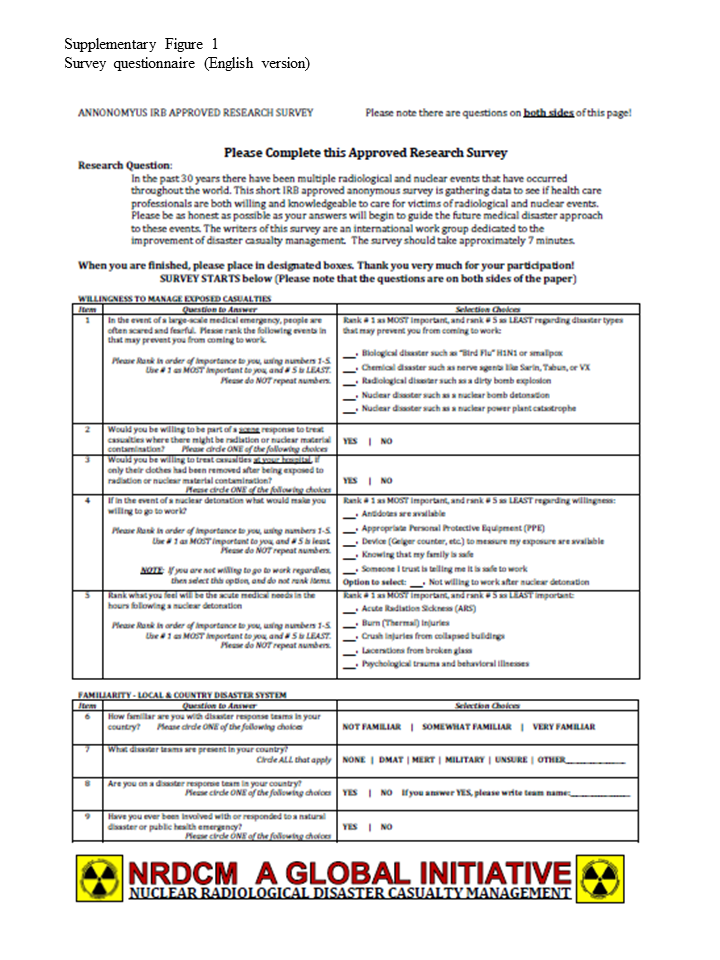

Supplement: Supplementary file 1 [file Image_1.TIF]

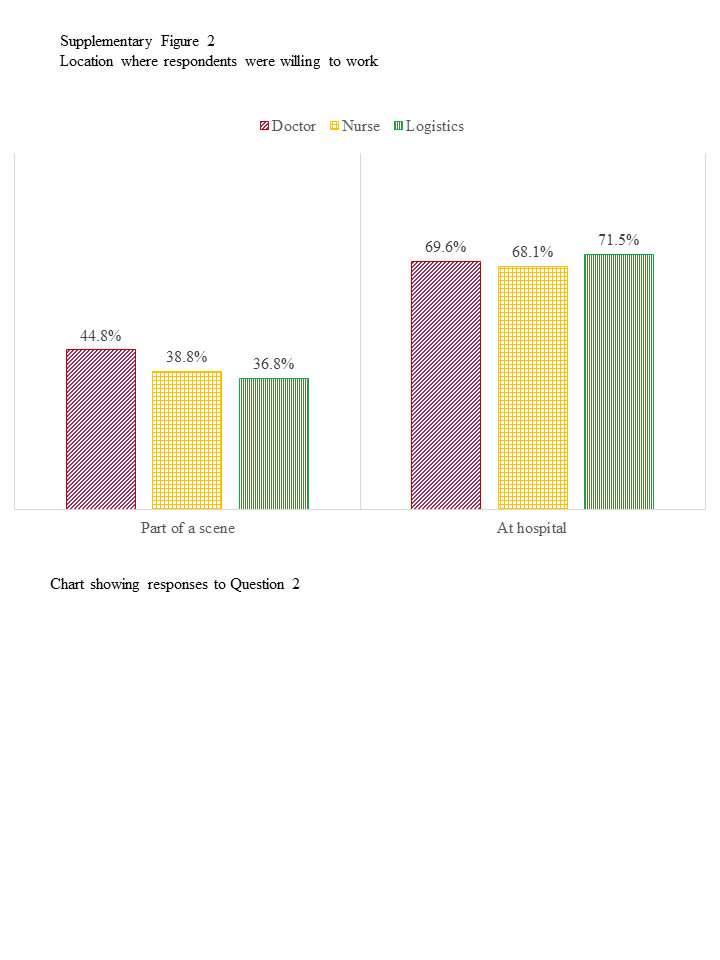

Supplement: Supplementary file 2 [file Image_2.tif]
